# Characteristics of Shigatoxin-Producing *Escherichia coli* Strains Isolated during 2010–2014 from Human Infections in Switzerland

**DOI:** 10.3389/fmicb.2017.01471

**Published:** 2017-08-03

**Authors:** Lisa Fierz, Nicole Cernela, Elisabeth Hauser, Magdalena Nüesch-Inderbinen, Roger Stephan

**Affiliations:** ^1^Institute for Food Safety and Hygiene, Swiss National Centre for Enteropathogenic Bacteria and Listeria, Vetsuisse Faculty, University of Zurich Zurich, Switzerland; ^2^National Reference Laboratory for Escherichia coli, Department of Biology Safety, Federal Institute for Risk Assessment Berlin, Germany

**Keywords:** shiga toxin producing *E. coli*, human infection, serogroups, *stx* subtypes, multilocus sequence types

## Abstract

**Objectives:** The aim of this study was to characterize a collection of 95 Shigatoxin-producing *E*.coli (STEC) isolated from human patients in Switzerland during 2010–2014.

**Methods:** We performed O and H serotyping and molecular subtyping.

**Results:** The five most common serogroups were O157, O145, O26, O103, and O146. Of the 95 strains, 35 (36.8%) carried *stx1* genes only, 43 strains (45.2%) carried *stx2* and 17 (17.9%) harbored combinations of *stx1* and *stx2* genes. *Stx1a* (42 strains) and *stx2a* (32 strains) were the most frequently detected *stx* subtypes. Genes for intimin (*eae*), hemolysin (*hly*), iron-regulated adhesion (*iha*), and the subtilase cytotoxin subtypes *subAB1, subAB2-1, subAB2-2*, or *subAB2-3* were detected in 70.5, 83.2, 74.7, and 20% of the strains, respectively. Multilocus sequence typing assigned the majority (58.9%) of the isolates to five different clonal complexes (CC), 11, 32, 29, 20, and 165, respectively. CC11 included all O157:[H7] and O55:[H7] isolates. CC32 comprised O145:[H28] isolates, and O145:[H25] belonged to sequence type (ST) 342. CC29 contained isolates of the O26:[H11], O111:[H8] and O118:[Hnt] serogroups, and CC20 encompassed isolates of O51:H49/[Hnt] and O103:[H2]. CC165 included isolates typed O80:[H2]-ST301, all harboring *stx2d, eae*-ξ, *hly*, and 66.7% additionally harboring *iha*. All O80:[H2]-ST301 strains harbored at least 7 genes carried by pS88, a plasmid associated with extraintestinal virulence. Compared to data from Switzerland from the years 2000–2009, an increase of the proportion of non-O157 STEC infections was observed as well as an increase of infections due to STEC O146. By contrast, the prevalence of the highly virulent German clone STEC O26:[H11]-ST29 decreased from 11.3% during 2000–2009 to 1.1% for the time span 2010–2014. The detection of O80:[H2]-ST301 harboring *stx2d, eae*-ξ, *hly, iha*, and pS88 related genes suggests an ongoing emergence in Switzerland of an unusual, highly pathogenic STEC serotype.

**Conclusions:** Serotyping and molecular subtyping of clinical STEC demonstrate that although STEC O157 predominates among STEC isolated from diseased humans, non-O157 STEC infections are increasing in Switzerland, including those due to STEC O146:[H2/H21/H28]-ST442/ST738 harboring *subAB* variants, and the recently emerged STEC O80:[H2]-ST301 harboring *eae*-ξ and pS88 associated extraintestinal pathogenic virulence genes.

## Introduction

Shiga toxin (Stx)-producing *Escherichia coli* (STEC) are important foodborne pathogens and responsible for outbreaks and sporadic cases of gastrointestinal illnesses which may include nonbloody or bloody diarrhea, hemorrhagic colitis (HC), and the hemolytic uremic syndrome (HUS) (Karch et al., 2005). Human pathogenic STEC produce one or more Stx, which consist of two groups designated Stx1 (consisting of the three variants Stx1a, Stx1c and Stx1d) and Stx2 (composed of seven distinct variants Stx2a, Stx2b, Stx2c, Stx2d, Stx2e, Stx2f, and Stx2g). Among these variants, Stx2a, Stx2c, and Stx2d are associated with severe disease and Stx2b and Stx2e are linked to mild clinical symptoms or asymptomatic fecal carriage (Stephan and Hoelzle, 2000; Friedrich et al., 2002; Fuller et al., 2011).

Furthermore, STEC strains may feature additional virulence traits that influence their pathogenic potential, such as intimin, enterohemolysin, adhesin, and subtilase cytotoxin (SubAB), which are encoded by *eae, hlyA, iha*, and *subAB* genes, respectively (Paton et al., 2004; Johnson et al., 2006; Käppeli et al., 2011a). *E. coli* O157:H7 is the serotype most frequently associated with outbreaks and severe clinical outcomes and is to date reported as the most common STEC serotype in the European Union and in Switzerland (Käppeli et al., 2011b; EFSA, 2016). However, non-O157 STEC serogroups, in particular, O26, O103, O111, and O145, are also recognized for their pathogenic potential and constitute together with O157 the so called “top five” serogroups of human pathogenic STEC in the EU (Beutin, 2006; Johnson et al., 2006; EFSA, 2016). Beside this group of five, other STEC serogroups, such as O91 and O121 have been associated with human illness in Germany and Switzerland, respectively (Mellmann et al., 2009; Käppeli et al., 2011a), and O45 and O121 are among the top seven serogroups detected in the U.S.A. (Gould et al., 2013). Changes in the Stx or serotype distribution of STEC infection is of public health significance as it may indicate the introduction of increasingly dominant strains. Identification of such strains is important as it may predict epidemiological changes or indicate novel sources of infection.

The aim of the present study was therefore to characterize all STEC strains collected by the Swiss National Centre for Enteropathogenic Bacteria and *Listeria* (NENT, Zurich, Switzerland) during 2010–2014 and compare these results with earlier data from Switzerland investigated over the 10-year period 2000–2009 (Käppeli et al., 2011a,b).

## Materials and methods

### Strain collection

A total of 95 Shigatoxin-producing *Escherichia coli* (STEC) strains isolated from human patients during 2010–2014 in Switzerland were characterized. The strain collection was obtained from the Swiss National Centre for Enteropathogenic Bacteria and Listeria (NENT). Forty-eight strains (50.5%) were from female and 47 strains (49.5%) from male patients. The median age was 24 years (range < 1–79 years). Forty-one strains (43.2%) were isolated from patients ≤ 5 years of age. The strains had all been confirmed to possess *stx* genes (*stx1* and/or *stx2*) by real-time PCR (LightCycler® 2.0 Instrument, Roche Diagnostics Corporation, Indianapolis, IN, USA), (EURL, 2013a).

### Serotyping

Strains were examined by PCR for the presence of genes associated with 15 selected serogroups including the top-five serogroups, namely O26, O45, O51, O55, O80, O91, O103, O104, O111, O113, O121, O128, O145, O146, and O157 (Perelle et al., 2004; EURL, 2013a,b, 2014; Soysal et al., 2016).

Strains belonging to other O groups were serotyped at the National Reference Laboratory for *Escherichia coli*, Federal Institute for Risk Assessment, Berlin, Germany, using standard methods and O–specific rabbit antisera. H types were determined by PCR, except for O51:H49, which had been typed previously using standard methods (Fasel et al., 2014). Strains were tested for the presence of flagellar genes related to H2, H4, H7, H8, H10, H11, H19, H21, H25, and H28 (Mora et al., 2012; EURL, 2013b; Beutin et al., 2015; Alonso et al., 2017).

### Virulence factor genes

The determination of *stx1* subtypes (*stx1a, stx1c, stx1d*) and *stx2* subtypes (*stx2a, stx2b, stx2c, stx2d, stx2e, stx2f*, and *stx2g*) was performed by conventional PCR amplification (Scheutz et al., 2012). Furthermore, the strains were screened by conventional PCR for *hlyA* (Schmidt et al., 1995), *iha* (Schmidt et al., 2001), and *subAB* (encoding SubAB), including its *subAB* subtypes (*subAB1, subAB2-1, subAB2-2*, and *subAB2-3*), as described previously (Tozzoli et al., 2010; Funk et al., 2013; Nüesch-Inderbinen et al., 2015; Müller et al., 2016; Tasara et al., 2017), using primers listed in Supplementary Table 1. Screening for *eae, aggR* coding for a transcriptional regulator in enteroaggregative *E. coli* (EAEC), and *elt* and *estIa/Ib* encoding heat-labile and heat stabile enterotoxins in enterotoxigenic *E. coli* (ETEC) was performed by real-time PCR according to the guidelines of the European Union Reference laboratory (European Union Reference (EURL, 2013c). Identification of the *eae* variants α1, α2, β1, γ1, γ2/θ, ε1, ζ, η, ι1, λ, and ξ was performed using primers described by Blanco et al. (2005).

Strains belonging to O80:H2 were screened by PCR for the pS88 related genes *sitA, cia, iss, iucC, iroN, hlyF, etsC, cvaA*, and *ompT*_*p*_, using primers described by Peigne et al. (2009).

### Further characterization of *E. coli* O157 strains

The collection of *E. coli* O157 strains was tested for sorbitol fermentation by using sorbitol MacConkey agar (SMAC) (Oxoid Ltd., Basingstoke, UK). All the *E. coli* O157 strains were analyzed by PCR for their flagellar (*fliC)* genotypes for *fliC*_H7_ as described previously (Gannon et al., 1997). The presence of O157 and the H7 antigen was corroborated with a latex agglutination test (Wellcolex™*E. coli* O157:H7, Remel, USA).

### Multilocus sequence typing (MLST)

MLST was performed by PCR amplification and sequencing of internal fragments of seven housekeeping genes (*adk, fumC, gyrB, icdF, mdh, purA*, and *recA*) (Wirth et al., 2006). Alleles and sequence types (STs) were assigned in accordance with the *E. coli* MLST database website (http://mlst.warwick.ac.uk/mlst/mlst/dbs/Ecoli).

## Results

### Identification of serotypes

Of the 95 STEC isolates, 78 (82.1%) were assigned to O types by PCR. Seventeen (17.9%) of the isolates did not fall into any of the serogroups tested for by PCR. Serological typing classified these strains into serogroups O46, O75, O76, O78, O80, O82, O84, O118, O156, O165, O166, O174, O177, O178, O183, ONT, and O rough. An overview of the determined serotypes is given in Table 1.

**Table 1:** Characteristics of 95 STEC strains isolated from human patients from 2010 to 2014 in Switzerland.

**Serotype^a^**	**No. of strains**	***stx*1**	***stx*2**	***eae***	***hlyA***	***iha***	***subAB***	***aggR***	***elt, estIa/Ib***	**ST**	**CC**
O26:[H11]	9	*stx1a*	–	β1	+	+	–	–	–	21	29
O26:[H11]	1	*stx1a*	*stx2a*	β1	+	+	–	–	–	29	29
O46:[Hnt]	1	*stx1a*	*stx2c*	–	+	+	–	–	–	154	–
O51:[Hnt]	1	–	*stx2a*	β1	–	–	–	–	–	20	20
O51:H49	1	–	*stx2e*	β1		–	–	–	–	20	20
O55:[H7]	1	–	*stx2a*	γ1		–	–	–	–	335	11
O75:[H25]	1	*stx1a*	*stx2a/stx2d*	–	+	+	–	–	–	3249	–
O76:[H19]	1	*stx1c*	*stx2b*	–	+	+	*subAB2-1/subAB2-2*	–	–	675	–
O78:[H7]	1	*stx1c*	–	–	+	+	*subAB2-1/subAB2-2*	–	–	3101	–
O80:[H2]	1	–	*stx2a*	ξ	+	+	–	–	–	301	165
O80:[H2]	2	–	*stx2d*	ξ	+	−	–	–	–	301	165
O80:[H2]	3	–	*stx2d*	ξ	+	+	–	–	–	301	165
O82:[Hnt]	1	*stx1a*	*stx2c*	–	+	+	*subAB1*	–	–	101	101
O84:[H2]	1	*stx1a*	–	ζ	+	+	–	–	–	306	–
O91:[H14]	1	*stx1a*	–	–	+	+	–	–	–	33	–
O91:[H14]	1	*stx1a*	*stx2b*	–	–	+	*subAB2-1/subAB2-2*	–	–	33	–
O91:[H10]	1	–	*stx2d*	–	−	+	–	–	–	641	86
O103:[H2]	9	*stx1a*	–	ε1	+	−	–	–	–	17	20
O103:[H2]	1	*stx1a*	–	ε1	+	+	–	–	–	386	20
O111:[H8]	1	*stx1a*	–	γ2/θ	+	+	–	–	–	16	29
O113:[H4]	1	*stx1c*	*stx2b*	–	+	+	*subAB2-1/subAB2-2*	–	–	10	10
O113:[H4]	1		*stx2b*	–	+	+	*subAB2-1/subAB2-2*	–	–	10	10
O118:[Hnt]	1	*stx1a*	–	β1	+	+	–	–	–	21	29
O121:[H19]	1	–	*stx2a*	ε1	+	−	–	–	–	655	–
O145:[H28]	7	–	*stx2a*	γ1	+	+	–	–	–	32	32
O145:[H25]	1	*stx1a*	–	γ1	+	−	–	–	–	342	–
O145:[H25]	1	*stx1a*	*stx2a*	γ1	+	−	–	–	–	342	–
O145:[H25]	2	–	*stx2a*	γ1	+	–	–	–	–	342	–
O145:[H25]	1	–	*stx2a/stx2c*	γ1	+	+	–	–	–	342	–
O146:[H2]	1	*stx1c*	–	–	+	+	*subAB2-1/subAB2-2*	–	–	442	–
O146:[H21]	1	*stx1c*	–	–	–	+	*subAB2-1/subAB2-2*	–	–	442	–
O146:[H21]	1	*stx1c*	*stx2b*	–	+	+	*subAB2-1/subAB2-2*	–	–	442	–
O146:[H28]	1		*stx2b*	–	+	–	*subAB2-1/subAB2-3*	–	–	738	–
O146:[H28]	3		*stx2b*	–	–	+	*subAB2-2*	–	–	738	–
O146:[H28]	1		*stx2b*	–	–	+	*subAB2-3*	–	–	738	–
O156:[H25]	1	*stx1a*	–	ζ	+	–	–	–	–	4942	–
O156:[H25]	1	*stx1a*	–	nt	+	–	–	–	–	5343	–
O157:[H7]	1	*stx1a*	–	γ1	+	+	–	–	–	11	11
O157:[H7]	2	*stx1a*	*stx2a*	γ1	+	+	–	–	–	11	11
O157:[H7]	4	*stx1a*	*stx2c*	γ1	+	+	–	–	–	11	11
O157:[H7]	4	–	*stx2a*	γ1	+	+	–	–	–	11	11
O157:[H7]	5	–	*stx2a/stx2c*	γ1	+	+	–	–	–	11	11
O157:[H7]	2	–	*stx2c*	γ1	+	+	–	–	–	11	11
O165:[H25]	1	–	stx2a/stx2c	ε1	+	–	–	–	–	119	–
O166:[H28]	1	*stx1c*	–	–	+	+	*subAB2-2*	–	–	1819	–
O174:[H28]	1	*stx1c*	–	–	–	+	*subAB2-1*	–	–	13	13
O174:[Hnt]	1	*stx1a*	*stx2d*	–	+	+	–	–	–	661	–
O174:[H21]	1	–	stx2c	–	–	+	–	–	–	677	–
O177:[H7]	1	*stx1a*	–	–	–	+	–	–	–	504	–
O178:[H19]	1	–	stx2c	–	–	+	–	–	–	192	–
O183:[Hnt]	1	*stx1a*	*stx2a*	–	+	+	–	–	–	657	–
ONT:[H21]	1	*stx1c*	–	–	+	+	*subAB2-1/subAB2-2*	–	–	737	–
ONT:[Hnt]	1	–	*stx2a*	–	–	–	–	–	–	10	10
ONT:[H7]	1	–	*stx2b*	–	–	+	*subAB2-2*	–	–	415	59
O rough:[Hnt]	1	*stx1c*	–	–	+	+	*subAB2-1/subAB2-2*	–	–	278	–

a *H types determined by PCR are denoted in square brackets ([H])*.

*aggR gene, encoding transcriptional regulator in enteroaggregative E. coli; CC, clonal complex; eae, intimin gene; elt and estIa/Ib genes, encoding heat-labile and heat stabile enterotoxins in enterotoxigenic E. coli; hlyA, hemolysin gene; iha, encoding iron-regulated adhesin gene; nt, not typable; ST, sequence type; stx, Shiga toxin gene (and subtypes); STEC, Shiga toxin-producing Escherichia coli; subAB, subtilase cytotoxin gene (and subtypes); +, the gene is present; –, the gene is absent; -, not applicable*.

Among the 95 isolates, 18 (19%) were O157:[H7], and 77 (81%) were non-O157 STEC isolates. Of the non-O157 strains, O145:[H25/H28] was the most common (*n* = 12/15.6% of all non-O157 isolates), followed by O26:[H11] (*n* = 10/13%), O103:[H2] (*n* = 10/13%), and O146:[H2/H21/H28] (*n* = 8/10.4%).

### Detection of virulence genes

Of the 95 STEC strains, 35 (36.8%) carried *stx1* genes only: *stx1a* (*n* = 28) and *stx1c* (*n* = 7). Forty-three strains (45.2%) carried *stx2* genes only: *stx2a* (*n* = 18), *stx2b* (*n* = 7), *stx2c* (*n* = 4), *stx2d* (*n* = 6), *stx2e* (*n* = 1), and *stx2a*/*stx2c* (*n* = 7). Seventeen strains (18%) harbored combinations of *stx1* and *stx2* genes. Forty-eight strains (50.5% of all isolates) carried the subtypes associated with high pathogenic potency, *stx2a, stx2c*, or *stx2d* (Table 1). The majority thereof (*n* = 29/60.4% of the 48 strains) belonged to the top-five serogroups, predominantly to O157 (*n* = 17) and O145 (*n* = 11), but notably, six (12.5%) belonged to serogroup O80.

Twelve isolates harbored the low pathogenic subtypes *stx2b* and *stx2e* and were mainly associated to the serogroups O146 (*n* = 6/50% of the 12 isolates), as shown in Table 1. None of the isolates harbored *stx2f*.

Genes for intimin, enterohemolysin, iron-regulated adhesion and subtilase cytotoxin were detected in 67 strains (70.5% of all isolates), 79 strains (83.2%), 71 strains (74.7%), and 19 strains (20%), respectively. The majority of the *subAB* harboring isolates (*n* = 11/57.9%) was associated with *stx2b* (Table 1).

The 67 *eae* positive isolates comprised six intimin variants including β1 (*n* = 14/20.9% of the 67 isolates), γ1 (*n* = 31/46.3%), and ε1 (*n* = 12/17.9%), ξ (*n* = 6/9%), ζ (*n* = 2/3%), γ2/θ (*n* = 1/1.5%) and one (1.5%) non-typeable (nt) strain (Table 1).

Of the six O80:[H2]-ξ strains, all (100%) carried pS88-related genes *sitA, cia, iss, iroN, hlyF, cvaA*, and *ompT*_*p*_, and five (83.3%) additionally had the *iucC* and the *etsC* genes (data not shown).

All 95 STEC strains were negative for *aggR, elt* and *estIa/Ib*.

### Further characterization of the O157-positive strains

Sixteen (88.9% of all O157-positive isolates) were non-sorbitol fermenters (nSF) on SMAC. Thereof, 11 were O157:H7 by latex agglutination. Two strains (11.1%) fermented sorbitol (SF), both were O157:H- by latex agglutination. However, all 18 strains tested positive for *fliC*_H7_ by PCR (Table 1).

### Multilocus sequence typing

An overview of the sequence types of the isolates is given in Table 1.

MLST assigned the majority (58.9%) of the isolates to 5 different clonal complexes (CC): CC11 (*n* = 19), CC20 (*n* = 12), CC29 (*n* = 12), CC32 (*n* = 7), and CC165 (*n* = 6).

Isolates of O serogroup O157 and O55 clustered in CC11. Isolates of O103 belonged to ST17 or ST386 and were all found with O51, within CC20. Isolates of the serogroups O26 (ST21 and ST29), O111 (ST16) and O118 (ST21) clustered within CC29. Seven of the 12 O145 isolates clustered in CC32, whereas further five O145 isolates belonged to ST342 and were not assigned to any CC. The isolates of serogroup O80 belonged to ST301 and CC165.

## Discussion

This study describes the serotypes, virulence genes and multilocus sequence types of STEC associated with human disease in Switzerland during 2010 and 2014. The five most common serogroups were O157, O145, O26, O103, and O146, with *E. coli* O157 accounting for 19% of the STEC-related infections. By comparison, during 2000 and 2009, 30.6% of the STEC strains isolated from clinical cases in Switzerland were STEC O157 (Käppeli et al., 2011b). Thus, as reported for other countries in the EU (ECDC, 2015), the proportion of non-O157 STEC associated with human STEC infections has increased also in Switzerland.

We further observed a change with regard to the most common non-O157 serotypes reported for the time span of 2000–2009 in Switzerland, O26:H11, O103:H2, O121:H19, and O145:H28/H^−^), respectively (Käppeli et al., 2011a). Compared with the previous study period, infections due to STEC O121:[H19] decreased (from 6.2 to 1.3% of all non-O157 STEC infections), while those accounted for by STEC O146:H2/21 and STEC O146:H28 increased (from 0 to 3.9%, and 1 to 5.2%, respectively) during 2010–2014. Evaluation of the virulent characteristics of the STEC serotypes showed that the majority of the common serogroups O157, O145, and O26 harbored *stx2a* alone or in combination with *stx1* or *stx2c*, and showed a consistent *eae* and *hlyA* gene pattern as described previously (Käppeli et al., 2011a,b).

Intimin γ1 was detected most frequently in this study and was associated with the most frequent STEC strains O157:[H7| and O145:[H28/[H25], in agreement with previous observations for STEC from humans (Beutin et al., 2004). Similarly, intimin β1 was found predominantly in STEC O26:[H11] and intimin ε1 in O103:[H2] and O121:[H19] (Beutin et al., 2004). Notably, the rare intimin variant ξ was associated with serotype O80:[H2].

Of the five most common serogroups found in this study, STEC O146 was the only serogroup that lacked the *eae* gene, but contained *subAB* genes. Subtilase cytotoxin SubAB is an emerging pathogenic factor that it is not routinely searched for in isolates from patients with STEC infections. Among the 95 isolates analyzed in this study, 67.9% of the *eae* negative strains harbored one or more *subAB* subtypes, including the recently described *subAB2-3* (Nüesch-Inderbinen et al., 2015) and 42.1% of all *subAB*-positive strains were STEC O146. Whereas *subAB1, subAB2-1*, and *subAB2-2* have been detected in clinical isolates elsewhere (Paton et al., 2004; Khaitan et al., 2007; Hoang Minh et al., 2015; Son et al., 2015), this is to our knowledge the only human clinical isolate harboring *subAB2-3* described so far. Its genome sequence is available under GenBank accession number MPGQ00000000 (Tasara et al., 2017). Our results indicate that the presence of *subAB* among STEC associated with human disease may be currently underestimated. However, this study was limited by the lack of anamnestic data to allow a correlation of the presence of SubAB with severity of disease.

The five most common serogroups in this study belonged to a limited number of CC and ST, whereas the vast majority of the remaining STEC strains were represented by a total of 28 STs, 24 of which contained only a single isolate. CC11 comprised all the O157 STEC, including the SF strains, confirming high clonality of this serogroup as described previously (Bielaszewska et al., 2007; Kossow et al., 2016). Furthermore, this CC included an isolate belonging to O55, which is in accordance to its evolutionary relatedness to O157 (Bono et al., 2012).

CC20 included all the O103 STEC analyzed in this study, indicating a monophyletic origin of these strains. MLST further demonstrated that O103 and O51 clustered together in CC20, suggesting clonal relationship of these isolates.

Within CC29, only one of the isolates was O26:[H11]-ST29. STEC O26:[H11] belonging to this particular ST and harboring *stx1a*/*stx2a* or *stx2a* alone has been described as a virulent clone that emerged in Germany and has been circulating in Europe since the mid-1990ies (Bielaszewska et al., 2013). Whereas during 2000–2009, O26:H11-ST29 harboring *stx1a*/*stx2a* or *stx2a* alone was detected in 11.3% of human infections due to STEC in Switzerland (Käppeli et al., 2011a; Zweifel et al., 2013), in the time span 2000–2014 only one such strain (1.1% of all isolates) was isolated, suggesting a decline of the highly virulent German clone in Switzerland over the last decade. On the other hand, the percentage of STEC O26:[H11]-ST21, which during 2000 and 2009 accounted for 57.1% of the STEC O26 infections, increased to 90% for the time span under observation. This phenomenon has public health relevance, since all (100%) of the STEC O26:[H11]-ST21 harbored *stx1a*, which is associated with milder course of disease. Multilocus sequence typing further revealed that isolates of O26, O111, and O118 clustered together in the same CC, indicating close relationship of these serogroups, as established previously (Ju et al., 2012; DebRoy et al., 2016).

CC32 contained seven isolates of the serogroup O145 ST32. In contrast, five further O145 isolates were ST342, which differs in its allelic profile in all loci from ST32. This indicates that the STEC O145 isolates originate from different clonal sources. Isolates belonging to STEC O146 fell into two major STs (442 and 738, respectively), neither of which were allocated to a particular CC.

Finally, 6.3% of the isolates analyzed in this study typed O80:[H2]-ST301 and clustered within CC165. All these isolates (100%) harbored *stx2 (stx2a or stx2d), eae*-ξ, and *hlyA*, 66.7% harbored *iha*, and 83.3% carried 7 or more pS88 related genes, revealing the potential of this serogroup as an etiological agent of severe infections. O80 is a serogroup that is difficult to type and may have gone under-detected and under-reported so far. Recently, however, O80:H2-ST301-ξ has been reported associated with HUS and bacteremia in France (Soysal et al., 2016). Further studies are warranted to elucidate the reservoirs and transmission routes of this unusual STEC.

## Conclusions

We describe a collection of 95 clinical STEC strains based on O and H serotyping, multilocus sequence typing and molecular subtyping of virulence genes, including *stx* and *eae* subtyping and screening for *subAB* variants. STEC isolated during 2010–2014 were distinguished by the presence of O157:[H7|-ST11-γ1, O145:[H28/[H25]-ST32/ST342-γ1, O26:[H11]-ST21-β1, O103:[H2]-ST20-ε1, and *eae*-negative O146:[H2/H28]-ST442/ST738 harboring *subAB* variants. Furthermore, we suggest that O80:[H2]ST-301-ξ, an STEC that possesses a rare intimin variant and a high extraintestinal virulence potential due to the presence of plasmid pS88-associated genes is emerging in Switzerland. Continued efforts are required to elucidate the origins and dissemination of this unusual STEC.

## Author contributions

RS and EH designed the study. LF, MN, and RS analyzed and interpreted the data. LF and NC carried out the microbiological and molecular biological tests. LF and MN drafted the manuscript. All authors read and approved the final manuscript.

### Conflict of interest statement

The authors declare that the research was conducted in the absence of any commercial or financial relationships that could be construed as a potential conflict of interest. The reviewer AMG and handling Editor declared their shared affiliation, and the handling Editor states that the process nevertheless met the standards of a fair and objective review.
