# Supplementary material for: Characteristics of Shigatoxin-Producing Escherichia coli Strains Isolated during 2010–2014 from Human Infections in Switzerland
Source: Front Microbiol. 2017 Aug 3;8:1471. doi: 10.3389/fmicb.2017.01471 (PMC5540938; doi:10.3389/fmicb.2017.01471)
Supplement: Supplementary file 1 [file Table1.docx]

***Supplementary Material***

**Characteristics of Shigatoxin-producing *Escherichia coli* strains isolated during 2010-2014 from human infections in Switzerland**

Lisa Fierz , Nicole Cernela , Elisabeth Hauser , Magdalena Nüesch-Inderbinen, Roger Stephan*****

*** Correspondence:** stephanr@fsafety.uzh.ch

# Supplementary Table

Supplementary Table 1: PCR primers used in this study for targeting virulence genes *eae*, *hlyA*, *iha*, and *subAB* variants by conventional

PCR.

| Primer ID | Nucleotide sequence (5'–3') | Target | Amplicon  (bp) | Description  of gene product | References |
| --- | --- | --- | --- | --- | --- |
| hlyA 1 | GGT GCA GCA GAA AAA GTT GTA | *hlyA* | 1,551 | Enterohemolysin | (Schmidt et al. 1995) |
| hlyA 4 | TCT CGC CTG ATA GTG TTT GGT A |  |  |  | (Schmidt et al. 1995) |
| iha-I | CAG TTC AGT TTC GCA TTC ACC | *iha* | 1,305 | Iron-regulated adhesin | (Schmidt *et al.* 2001) |
| iha-II | GTA TGG CTC TGA TGC GAT G |  |  |  | (Schmidt *et al.* 2001) |
| subAB-V-for | CTT CCC TCA TTG CCT CAC G | *subAB* | 1,066 | Subtilase cytotoxin | (Funk *et al.* 2013) |
| subAB-V-rev | GGC TGG CCT GTT GTG TAA A |  |  |  | (Funk *et al.* 2013) |
| subAB-for5 | CGT ATC TGC GCC ATA TCC TG | *subAB1* | 1,820 | Subtilase cytotoxin | (Funk *et al.* 2013) |
| subAB-rev5 | CTG TTC CGA GCA GCC ATA TC |  |  |  | (Funk *et al.* 2013) |
| tia_lo | TCC ATG CGA AGT TGT TAT CA | *subAB2-1* | 3,174 | Subtilase cytotoxin | (Tozzoli *et al.* 2010) |
| subAB2-3′tia | ACT GGC TGT TCT AAC CG |  |  |  | (Funk *et al.* 2013) |
| subAB_out | GAATCAACAACAGATACGAC | *subAB2-2* | 1,746 | Subtilase cytotoxin | (Funk *et al.* 2013) |
| subAB5′OEP | TAA TGT TTT TGA GAC GGG |  |  |  | (Funk *et al.* 2013) |
| subAB_out | GAATCAACAACAGATACGAC | *subAB2-3* | 943 | Subtilase cytotoxin | (Funk *et al.* 2013) |
| subAB2-3rv | GAGGCGACTAATGAAGAATTAA |  |  |  | (Tasara *et al.* 2017) |
